# Single Muscle Immobilization Decreases Single-Fibre Myosin Heavy Chain Polymorphism: Possible Involvement of p38 and JNK MAP Kinases

**DOI:** 10.1371/journal.pone.0158630

**Published:** 2016-07-06

**Authors:** Frédéric Derbré, Mickaël Droguet, Karelle Léon, Samuel Troadec, Jean-Pierre Pennec, Marie-Agnès Giroux-Metges, Fabrice Rannou

**Affiliations:** 1 Laboratory “Movement Sport and health Sciences”(M2S) -EA1274, University Rennes 2-ENS Rennes, Rennes, France; 2 Physiology Department-EA1274 M2S, School of Medicine, Brest, France; Cinvestav-IPN, MEXICO

## Abstract

**Purpose:**

Muscle contractile phenotype is affected during immobilization. Myosin heavy chain (MHC) isoforms are the major determinant of the muscle contractile phenotype. We therefore sought to evaluate the effects of muscle immobilization on both the MHC composition at single-fibre level and the mitogen-activated protein kinases (MAPK), a family of intracellular signaling pathways involved in the stress-induced muscle plasticity.

**Methods:**

The distal tendon of female Wistar rat *Peroneus Longus* (PL) was cut and fixed to the adjacent bone at neutral muscle length. Four weeks after the surgery, immobilized and contralateral PL were dissociated and the isolated fibres were sampled to determine MHC composition. Protein kinase 38 (p38), extracellular signal-regulated kinases (ERK1/2), and *c*-Jun- NH2-terminal kinase (JNK) phosphorylations were measured in 6- and 15-day immobilized and contralateral PL.

**Results:**

MHC distribution in immobilized PL was as follows: I = 0%, IIa = 11.8 ± 2.8%, IIx = 53.0 ± 6.1%, IIb = 35.3 ± 7.3% and I = 6.1 ± 3.9%, IIa = 22.1 ± 3.4%, IIx = 46.6 ± 4.5%, IIb = 25.2 ± 6.6% in contralateral muscle. The MHC composition in immobilized muscle is consistent with a faster contractile phenotype according to the Hill’s model of the force-velocity relationship. Immobilized and contralateral muscles displayed a polymorphism index of 31.1% (95% CI 26.1–36.0) and 39.3% (95% CI 37.0–41.5), respectively. Significant increases in p38 and JNK phosphorylation were observed following 6 and 15 days of immobilization.

**Conclusions:**

Single muscle immobilization at neutral length induces a shift of MHC composition toward a faster contractile phenotype and decreases the polymorphic profile of single fibres. Activation of p38 and JNK could be a potential mechanism involved in these contractile phenotype modifications during muscle immobilization.

## Introduction

Adult skeletal muscle exhibits a remarkable ability to adjust contractile phenotype to functional demands. Such diversity of skeletal muscle to perform a variety of shortening speeds is due to the assemblage of different fibre types. A strong correlation exists between a fibre’s speed of shortening and its myosin heavy chain (MHC) isoform expression [1, 2]. Furthermore, at single-fibre level, mammalian skeletal muscle fibers can contain either one single major MHC (i.e. type I, IIa, IIx, and IIb) or a combination of different MHC isoforms (i.e. hybrid fibers). The functional significance of these hybrid fibres remains an unresolved issue. These mixed phenotype fibres have been interpreted has a fine functional tuning since their shortening velocities are intermediate between “pure” fibres [3, 4]. Further, fibres coexpressing different MHC isoforms are meant to allow a rapid switch to meet new functional needs in case of contractile phenotype modification.

It has previously been shown that skeletal muscle disuse leads to increase MHC polymorphism [5, 6]. For example, spinal cord transection increases the proportion of hybrid fibres within 15 days following the transection [7]. Muscle immobilization also leads to modification in the contractile properties of skeletal muscle [8–12]. Such modification in the contractile phenotype may be due to either a decrease in motor neuron discharges, the length of immobilization, and muscle shortening prevention. In order to circumvent these limitations, another model consists in the isolated immobilization of one muscle at neutral length [11, 13, 14]. In this model, neural inputs are preserved and muscle contraction occurs in an isometric manner [13]. After 4 weeks of immobilization, the time to peak force and the resistance to fatigue are reduced, corresponding to a faster contractile phenotype [11]. However, in this model, less is known about the effects of immobilization on MHC content and polymorphism at single-fibre level.

The regulation of MHC isoforms expression is controlled by different intracellular signaling pathways [2], involving among others the family of nuclear factor of activated T-cells (NFATc) and the mitogen-activated protein kinases (MAPK) signaling cascades [15–18]. NFATc family members play a specific role in fibre type specification in adult skeletal muscle [19]. The MAPK family consists of three parallel pathways that include protein kinase 38 (p38), extracellular signal-regulated kinases (ERK1/2), and *c*-Jun- NH2-terminal kinase (JNK). Interestingly, it has been demonstrated *in vitro* that modulation of subcellular localization of NFATc through p38, JNK and ERK1/2 constitutes a potential mechanism linking MAPK and MHC distribution [20]. More specifically, p38 and JNK would directly phosphorylate NFATc1 serine residues and inhibit the translocation of transcription factor to the nucleus, thus promoting reduction in MHCI protein content [20, 21].

From these data, we hypothesized that muscle immobilization would induce a faster muscle phenotype characterized by 1) an increase of single fibre expressing MHCIIx and IIb isoforms 2) an activation of MAPK associated with a reduction of single fibre expressing MHCI isoforms. Therefore, the present study was designed to evaluate the MHC composition of immobilized Peroneus Longus (PL) on single-fibre level. The PL contains the four types of MHC [22] and displays intermediate contractile properties between the typical “slow” and “fast” muscles, namely, Soleus and Extensor Digitorum Longus [23]. A second objective was to explore the effects of muscle immobilization on the MAP kinases signaling pathway, and their potential role on MHC composition.

## Materials and Methods

### Ethical Approval and Animal Care

**Fig 1:**
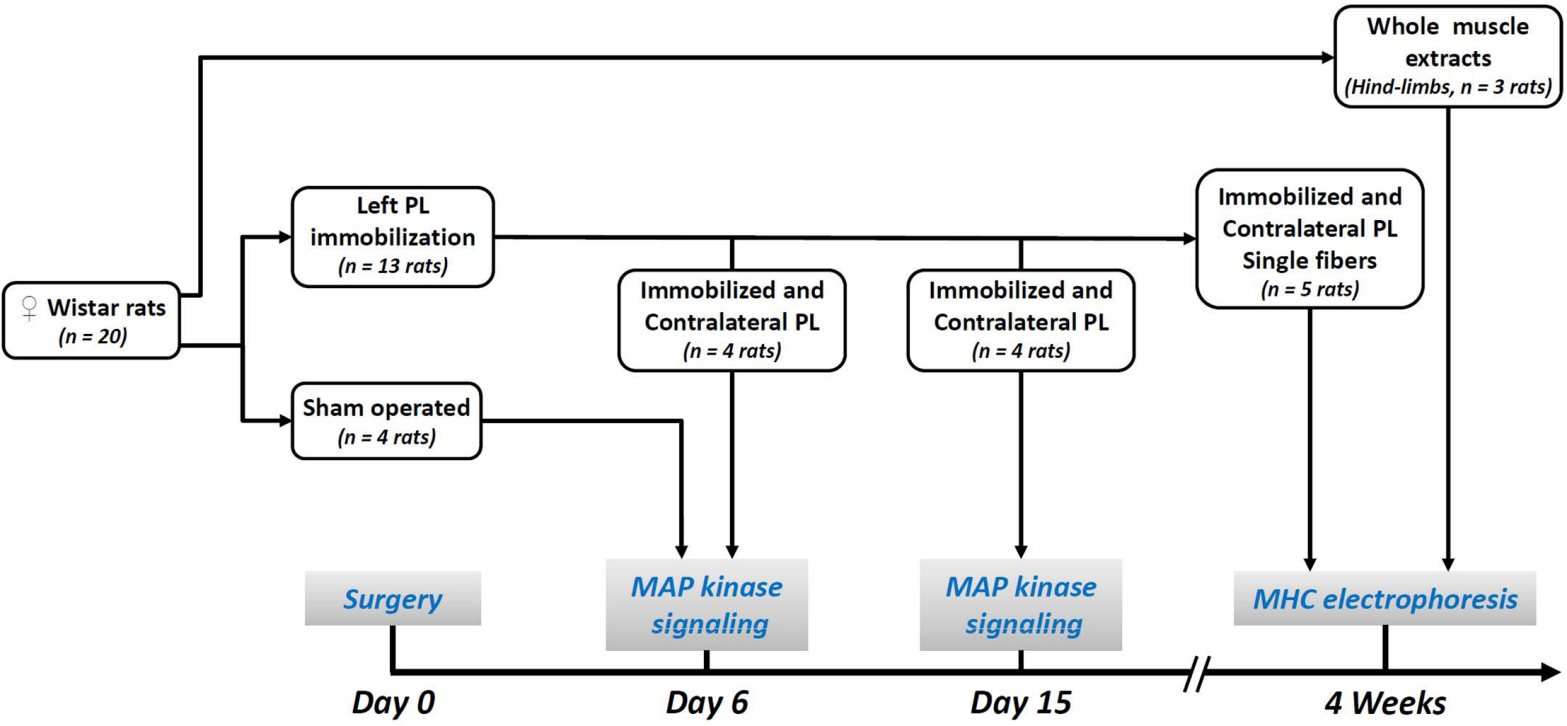
Experimental work-flow diagram. PL, *Peroneus Longus*; MAP Mitogen-Activated Protein; MHC, Myosin Heavy Chain.

The experiments were performed according to our ethical regional committee recommendations (experimental protocol No. 02076.01), and in compliance with the recommendations of European Community Directive No. 2010/63/EU. The procedures were authorized by the relevant ethical committee ("Comité d'éthique finistérien en expérimentation animale") with the departmental agreement No. A29-019-03. Female Wistar rats were purchased from Janvier (Le Genest Saint-Isle, France) and housed in a controlled condition of temperature and humidity with a 12-h light–dark cycle. Animals were provided with standard rodent diet (Kliba No. 3800, Provimi Kliba AG, Kaiseraugst, Switzerland) and water ad libitum. Twenty animals, aged 12.1 ± 1.3 weeks and weighing 260–280g, were randomly assigned to three groups as shown in Fig 1: immobilization (n = 13), sham (n = 4), migration marker for MHC electrophoresis (n = 3). Surgical procedures were performed under deep general anaesthesia (80 mg.kg^-1^ IP ketamine, Imalgene°, Mérial and 12 mg.kg^-1^ IP xylazine, Rompun°, Bayer) and under aseptic conditions. Buprenorphine (0.1 mg.kg^-1^ SC, Vetergesic°, Sogeval) was administered at the end of the surgery for analgesia.

### Muscle Immobilization

In 13 animals, the left Peroneus Longus (PL) was set to a neutral length corresponding to a 90° flexion of ankle joint. The distal tendon of left PL was cut and attached with a nylon thread (Prolène° 6.0) to a hole in the adjacent bone, as previously described [11, 13, 14]. The contralateral muscle was used as control [9, 24, 25]. After surgery, rats were randomly assigned to three groups (Fig 1): eight were euthanized for Western Blot, four at 6 days and four at 15 days after immobilization, and five animals were killed at postoperative week 4 for single fibre electrophoresis.

### Sham

To further evaluate the effects of the surgery on the intracellular signaling pathways of muscle, the peroneal tendon sheath of the left hindlimb was opened above the lateral malleolus and a hole was made in the adjacent bone (n = 4 animals). As in “immobilized” animals, the tendon sheath and the skin were sutured by using a double-layer procedure [11].

### Muscle Dissociation

Four weeks after the surgical procedure (n = 5 animals), single fibres from immobilized and contralateral PL were dissociated using enzymatic and mechanical methods [11, 22, 26]. Such methodology allows for the complete dissociation of PL, ensuring the sampling of isolated fibres from any muscle regions. After anaesthesia with pentobarbital (40 mg.kg^-1^ IP, Pentobarbital Sodique°, Ceva) and cervical dislocation, PL muscles were rapidly excised from the hindlimb. PL muscles were placed in a medium containing a standard buffered saline solution [150 mM NaCl, 5mM KCl, 2 mM CaCl_2_, 1 mM MgCl_2_, 30 mM N-2-hydroxyethylpiperazine-N = -2-ethanesulfonic acid (HEPES); pH = 7.4] supplemented with 3.0 mg.ml^-1^ collagenase (type II; GIBCO-BRL, Gaithersburg, MD) for 2 h at 37°C. After this incubation period, muscles were dissociated using gentle agitation. Isolated fibres were randomly sampled using automatic 10 μl pipette and transferred into 1.5 mL polypropylene tube (Eppendorf°, Hamburg, Germany) that contained 20 μl of denaturing lysis buffer (0.3 M NaCl, 0.1 M NaH_2_PO_4_, 50 mM Na_2_HPO_4_, 0.01 M Na_4_P_2_O_7_, 1 mM MgCl_2_.6H_2_O, 10 mM EDTA, 1.4 mM *β*−mercapto-ethanol) for 24 h at 4°C. After 12 h at 4°C, fibres were glycerinated with a 40% glycerol solution (5 μL) and stored at -80°C until electrophoresis.

### Single Fibre Electrophoresis

MHC isoforms were electrophoretically separated by the sodium dodecyl sulfate polyacrylamide gel electrophoresis (SDS-PAGE) method as previously described (adapted from [27], [22]). The single fibre extracts were loaded on high-glycerol-containing (30%) gels with an acrylamide-to-bis-acrylamide ratio of 50:1 for the separating gel (8% total acrylamide, pH 8.8) and for the stacking gel (4% total acrylamide, pH 6.8) using a Mini-Protean II dual slab cell apparatus (Bio-Rad, Hercules, CA). Electrophoresis was run for 22 h at 140 V in a refrigerated room at 4°C. Gels were silver stained (Silver Stain Plus kit, Biorad, Richmond, CA) and the different MHC isoforms (I, IIa, IIx, IIb) were identified according to their electrophoretic mobility pattern [22, 27]. In fibres expressing more than one MHC isoform, their relative abundance was determined using scanning densitometry (Mesurim Pro Software, jean-francois.madre@ac-amiens.fr). A polymorphism index for immobilized and contralateral PL was calculated as previously described [28]:

Polymorphism Index(%)=(no.of polymorphic fibres/total no.of fibres)×100

### Hill’s Statistical Model

In an attempt to provide a functional assessment of the variation of MHC distribution on muscle’s speed of shortening, the relative MHC content in whole immobilized and contralateral PL was determined from single-fibre data [28–30]. For each PL, data from all analysed fibres (pure and hybrid) were used to calculate the relative MHC content of the whole muscle. According to the model proposed by A.V. Hill [31], we used the equation proposed by Caiozzo et al. to calculate the maximal shortening velocity of PL [28]:

$$V_{\max}\mathrm{PL}\;=\;\left(f_{I}\;\times \;V_{\mathrm{max I}}\right)\;+\;\left(f_{\mathrm{IIa}}\;\times \;V_{\mathrm{max IIa}}\right)\;+\;\left(f_{\mathrm{IIx}}\;\times \;V_{\mathrm{max IIx}}\right)\;+\;(f_{\mathrm{IIb}}\;\times \;V_{\mathrm{max IIb}})$$

Where f_x_ is the proportion of each MHC isoform in PL, and V_max x_ is the maximal shortening velocity for a given MHC isoform (fibre length.s^-1^). Values from Bottinelli et al. [1] were used for V_max x_. In this model, Soleus (Sol) and Extensor Digitorum Longus (EDL) were used as representative slow-twitch and fast-twitch muscles, respectively [32].

### Western-Blot

MAP Kinases were studied in 6-day, 15-day immobilized PL, and Sham PL muscles (n = 4 animals in each condition). Following euthanasia (40 mg.kg^-1^ IP pentobarbital, Pentobarbital Sodique°, Ceva and cervical dislocation), operated and contralateral PL muscles were immediately removed, weighted, and placed in a tube cooled by immersion in liquid nitrogen.

### Cytosolic Protein Extraction for Immunoblot Analyses

Cytosolic protein extraction was performed from PL in cold lysis buffer containing 10 mM Tris-HCl, pH 7.4, 0.5 M sucrose, 50 mM NaCl, 5 mM EDTA, 30 mM Na_4_P_2_O_7_, 1% NP-40, 0.25% sodium deoxycholate, 50 mM NaF, 100 μM sodium orthovanadate and proteases inhibitors cocktail (Sigma P8340, 5 μl.ml^-1^). The samples were homogenized using a Polytron homogenizer at 4°C. Each sample was then incubated on ice for 30 min followed by 3 x 10 s of sonication. The homogenates were transferred to microcentrifuge tubes and centrifugated at 12,000 *g* for 12 min at 4°C. The protein concentration of the supernatant was determined by a Lowry assay using bovine serum albumin as standard. Samples were then diluted in SDS-PAGE sample buffer [50 mM Tris-HCl, pH 6.8, 2% SDS, 10% glycerol, 5% β-mercaptoethanol, and 0.1% bromophenol blue], and heated 5 min at 95°C until analyses.

### Western Immunoblot Analyses

Samples containing 60 μg of proteins were resolved on 12.5% SDS-PAGE. The proteins were transferred at 240 mA for 90 min onto a 0.2-μm nitrocellulose membrane. Membranes were blocked with 5% BSA or nonfat dry milk in TBST (Tris-buffered saline/0.05% Tween-20) for 1 h at room temperature. Membranes were incubated overnight at 4°C with appropriate primary antibodies: anti-p38 MAPK (1:1000, Cell Signaling), anti-phospho-p38 MAPK (1:1000, Cell Signaling), anti-phospho-JNK (1:200, Santa Cruz), anti-JNK (1:200, Santa Cruz), anti-phospho ERK1/2 (1:1000, Cell Signaling), anti- ERK1/2 (1:1000, Cell Signaling), and α-actin (1:700, Sigma Aldrich). Thereafter, membranes were incubated with a secondary antibody for 1 h at room temperature. Blots were washed with TBST and incubated with infrared dye-conjugated secondary antibodies (LI-COR, Lincoln, NE, USA). After washing, blots were captured using the Odyssey Imaging System (LI-COR, Lincoln, NE, USA). All blots were scanned, and densitometric analysis of the bands was conducted using GS-800 Imaging densitometer and QuantityOne software. Phosphospecific and total signals were normalized to α-actin. For each animal, protein expression in immobilized and sham PL was normalized to contralateral muscle.

### Statistical Analyses

The number of animals needed in each group was determined using a significance level of 0.05 and 80% power (two-sided tests), and based on the following rationale. A 40% polymorphism index was expected in contralateral PL according to unpublished data collected in our laboratory in control PL. We assumed a standard deviation of 4% for index of polymorphism as previously shown in rodent hindlimb muscles [33] and 5% for muscle MHC composition [32–33]. We estimated that five rats in each group would allow us to detect a 8 and 10% between-group difference in index of polymorphism and MHC composition, respectively. For MAP kinase signaling, we estimated that a minimum sample size of four rats in each group would be sufficient to detect a mean difference of 2 xSD.

Results are presented as mean ± SD. Polymorphism index and MHC distribution in immobilized and contralateral muscles were compared using two-tailed Student’s paired t-test. MAPK phosphorylations between immobilized and contralateral muscles were compared using a Mann-Whitney test in each group (Sham, day 6 and day 15). The ratio of MAPK phosphorylations between immobilized and contralateral muscles was also calculated, and then analyzed using an ANOVA on ranks (Kruskal–Wallis test). If main time effects were observed, the differences were localized using a post hoc Dunn’s test. For all statistical analyses, the level of significance was set at *P* < 0.05.

## Results

### Effects of Peroneus Longus Immobilization on Single Fibre MHC Distribution Composition

**Fig 2:**
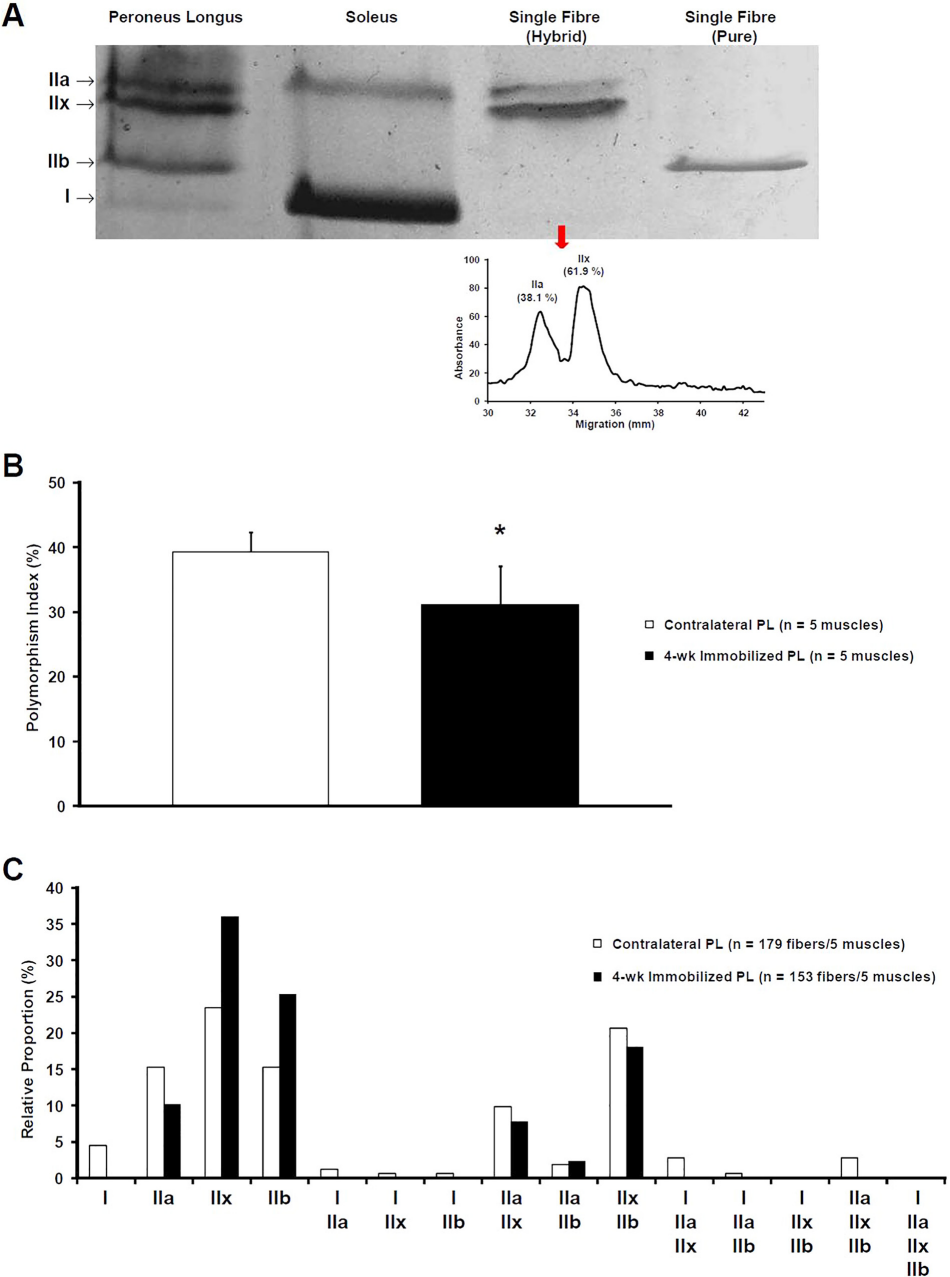
Effects of *Peroneus Longus* immobilization on the distribution of MHC in single muscle fibres. **A** Myosin heavy chain isoforms discriminated by 8% SDS-PAGE and silver staining. MHC isoforms present in single fibres from contralateral and 4-wk immobilized PL were identified by their migration pattern relative to known MHC composition from whole muscle extracts (PL and Soleus). In hybrid fibres, the relative amounts of the different MHC isoforms were determined using densitometric procedures. **B** The relative proportion of fibres expressing more than one MHC isoform was calculated for each 4-wk immobilized and contralateral PL. Data expressed as mean ± SD. * significantly different from contralateral PL (*P* = 0.028, Student’s paired t-test). **C** Analysis of the frequency distribution of each MHC-based fibre type in contralateral and 4-wk immobilized PL.

The effects of immobilization on the MHC pattern were evaluated at single-fibre level (Fig 2). Electrophoretic separation of MHC isoforms was performed on 153 and 179 single fibres of immobilized and contralateral PL, respectively. Four MHC bands were identified as type I (the fastest migrating band), IIb, IIx, and IIa (the slowest migrating band) by comparison with the migration patterns of whole-muscle extracts with known MHC composition. The relative number of hybrid fibres differed between immobilized and contralateral muscles. Hybrid fibres represented 31.1% (95% CI 26.1–36.0) and 39.3% (95% CI 37.0–41.5) of the analyzed fibres from immobilized and contralateral muscles, respectively (*P* = 0.028, Student’s paired t-test).

**Fig 3:**
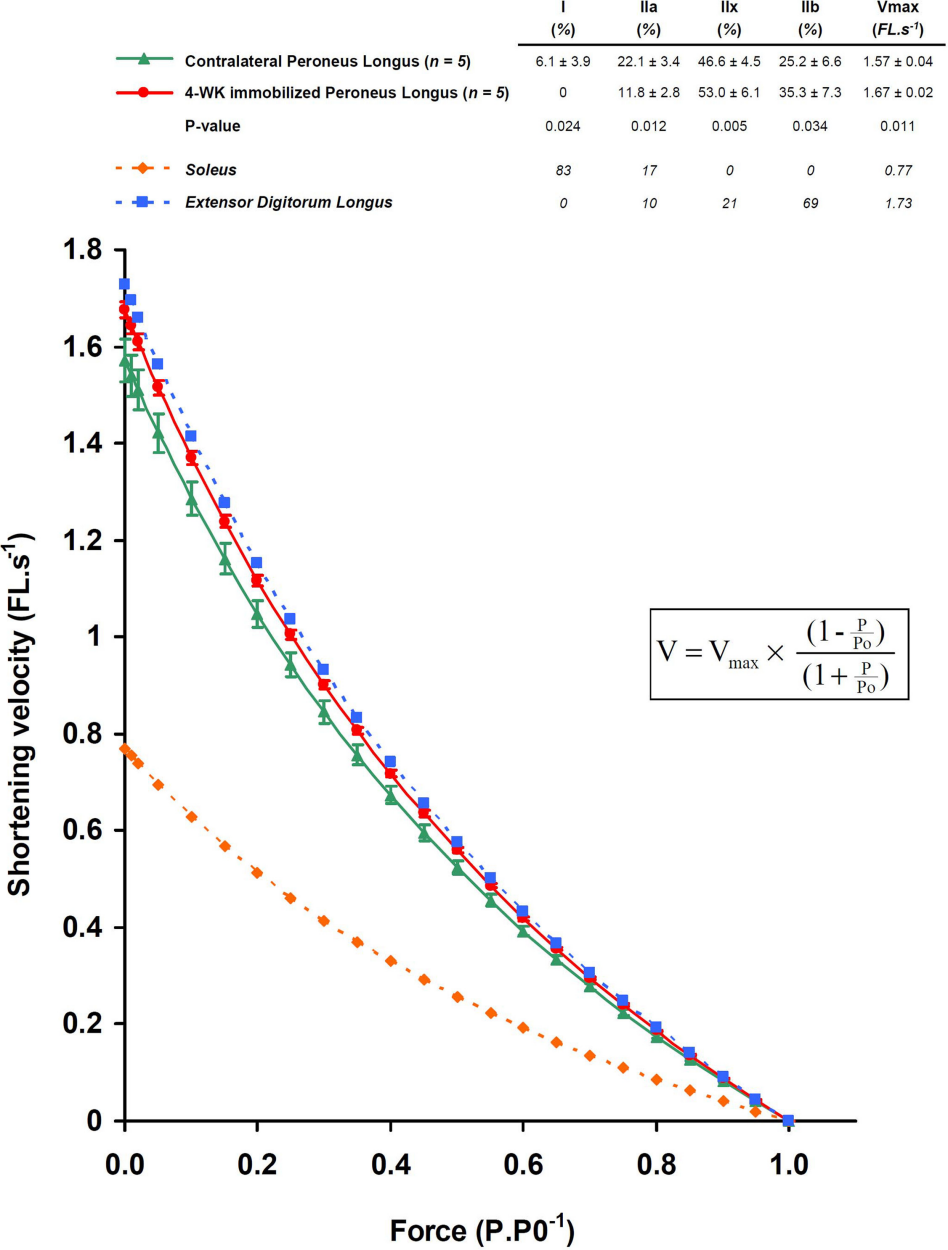
Prediction of the effects of immobilization on the force-velocity relationship. Whole muscle MHC isoform distribution in 4-wk immobilized and contralateral PL was determined from single-fibre analysis (pure and hybrid fibres), and compared to typical slow and fast muscles (Soleus and Extensor Digitorum Longus, respectively). Force-velocity curves were generated according to the Hill-type mathematical model (*inset*), by using the proportion of each MHC isoform in whole muscle. Data are expressed as mean ± SD. V, shortening velocity; V_max_, maximal shortening velocity; FL, fibre length; P, isotonic tension; Po, maximal isometric tension.

As shown in Fig 3, significant variations were observed within the four fibre types in immobilized PL. MHC IIx and IIb levels were higher in immobilized PL in comparison with contralateral muscle. The MHC I isoform was absent in fibres from immobilized muscles. The effects of immobilization on the force-velocity relationship of PL were determined according to the Hill’s characteristic equation using the relative content of fast and slow MHC isoforms in contralateral and immobilized PL. The predicted maximal shortening velocity of Control PL was 1.57 fibre length.s^-1^ (95% CI 1.53–1.61). As illustrated in Fig 3, a shift toward a faster phenotype was observed after 4 weeks of immobilization (1.67 fibre length.s^-1^, 95% CI 1.66–1.69; *P* = 0.011, Student’s paired t-test).

### Effects of Peroneus Longus Immobilization on MAPK Phosphorylation

**Fig 4:**
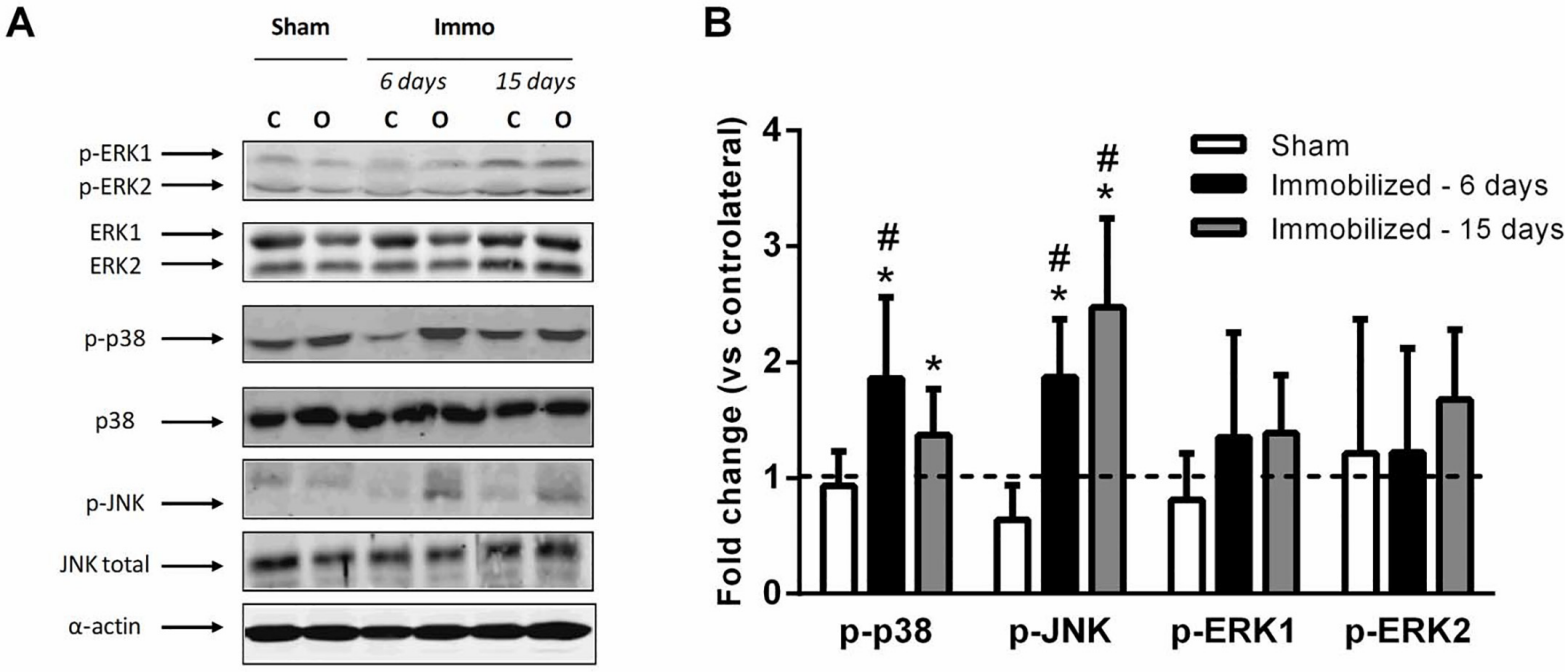
Activation of MAP kinases in skeletal muscle during immobilization. **A** Representative Western blot for phosphorylated and total ERK, p38, and JNK MAPK. **B** The MAP kinases phosphorylation in sham, 6- and 15-days immobilized PL were expressed relative to the contralateral muscle (n = 4 animals in each condition). C, contralateral; O, operated. Dashed line represents the basal levels in contralateral skeletal muscle. Error bars represent the SD. * MAPK activation significantly different from contralateral muscles (*P* < 0.05), # fold-change significantly different from sham-operated group (*P* < 0.05).

Fig 4 shows representative Western blots and relative contents of phosphorylated levels of MAP kinases in immobilized and contralateral PL. Baseline data of MAPK markers in immobilized and contralateral PL are presented in S1 Fig. We observed a higher p38 phosphorylation in immobilized vs. contralateral PL after 6 and 15 days of immobilization (1.9 ± 0.7 and 1.4 ± 0.4 fold respectively, *P* < 0.05, Fig 4B). Immobilization also increased JNK phosphorylation in immobilized PL after 6 and 15 days (1.8 ± 0.5 and 2.5 ± 0.8 fold respectively, *P* < 0.05, Fig 4B). In contrast with p38 and JNK, immobilization did not modulate ERK1 and ERK2 phosphorylation. In sham animals, MAPK activation was unchanged between operated and contralateral PL.

## Discussion

### Single Muscle Immobilization Induces a Transition from Slow-To-Fast Myosin Fibre Types

Myosin is the most abundant contractile molecule in mammalian skeletal muscles. In addition, numerous studies have shown that the primary determinant of single-fibre maximal shortening velocity is the MHC isoform (IIb>IIx>IIa>I). Thus, the modification in the proportion of MHC isoforms in skeletal muscle represents an important form of regulation of contractile properties. Approximately 31 and 36 single fibres per muscle (control and 4-wk immobilized PL, respectively) were randomly sampled and analysed, in line with previous studies [29, 30]. Using such methodology, it has been demonstrated that the MHC isoform composition of whole muscle is accurately predicted from the single-fibre analysis [29].

According to the MHC content, the predicted maximal shortening velocity of control PL lies in the mid-range of hindlimb muscle profiles [28], in agreement with previous measurements of the respective contractile properties of Soleus, PL, and EDL [23]. Disuse conditions (i.e. joint immobilization, hindlimb unloading, bed rest, spinal cord injury) are well recognized to induce a transition from slow-to-fast myosin fibre types in skeletal muscle [34–37]. This transition may be related to either or both reduction in motor neuron stimulation and in muscle length changes. Here, we used a different experimental model in which immobilization is performed by the fixation of the distal tendon of one single muscle to the adjacent bone. Neural inputs are preserved and the immobilized muscle remains at the same neutral length [13]. In accordance with other disused conditions, we observed an increase in muscle fibres expressing MHCIIx and MHCIIb with a concomitant reduction in muscle fibres expressing MHCI and MHCIIa isoforms. Our results and previous data support the view that the expressions of slow and fast MHC genes are regulated in an antithetic manner during muscle fibre type transitions [2, 38]. The absence of muscle stretching during the 4 weeks of immobilization could explain such reduction of MHCI isoforms in our model. Indeed, similar reduction is observed in other disuse conditions as hindlimb unloading, but this phenomenon is efficiently prevented when passive stretch is imposed to the “unloaded” muscle [39]. Moreover, electrostimulation during hindlimb unloading also prevents reduction in MHCI proportion in skeletal muscle [35, 40], indicating a key role of muscle shortening to maintain slow muscle phenotype. Consequently, these data indicate that the faster phenotype transition observed in disuse conditions is preferentially due to the absence of stretching or shortening rather than lower motor neuron stimulation.

In human, it should be emphasized that the conversion of skeletal muscle toward a faster contractile phenotype during disuse is paralleled by reduction in fibres cross-sectional area [37, 41, 42]. Interestingly, it has been proposed that the slow-to-fast conversion of MHC could contribute to counteract the atrophy-induced loss of force [43, 44]. During single-muscle immobilization, it has previously been shown that muscle atrophy is about 25.7% following four weeks of immobilization [11]. Thus, the present model is consistent with previous data in human model of disuse such as bed-rest [37, 42] or unilateral limb immobilization [41], that is the association of atrophy and slow-to-fast MHC conversion in skeletal muscle. The present experimental model is therefore suitable to study the complex interplay between quantitative (i.e. muscle mass) and qualitative (i.e. fibre type) modifications of skeletal muscle during disuse conditions.

During immobilization, modifications of the mechanical properties of the MHC isoform could also contribute to alter the contractile properties of skeletal muscle. In human, it has been shown that the shortening velocity of MHC-based fibre type is modified during disuse conditions [45]. However, it should be emphasized that results have been contradictory, with some studies reporting an increase and others a decrease in the shortening velocity of single fibres (see table 1 in [46]). Further studies using in-vitro measurements of the shortening velocity of “immobilized” fibres are needed to precise the contribution of this additional factor of plasticity in the present model.

### Single Muscle Immobilization Decreases the Polymorphism of Single Fibres

Adult skeletal muscles are featured by many hybrid fibres expressing more than one MHC isoform. It should be highlighted that marked variation in the proportion of hybrid fibres is observed in normal adult skeletal muscles [28]. As previously reported in hindlimb muscles [28, 47], the predominant hybrid fibres in control PL were IIa/IIx and IIx/IIb. In this study, fibre-to-fibre analysis showed that single-muscle immobilization leads to decrease the proportion of hybrid fibres. The physiological relevance of single fibre polymorphism in adult skeletal muscle is a matter of debate, and a unifying model is still lacking. It has been proposed that hybrid fibres represent fibres in a transitional state between pure types [47]. Nevertheless, it should be emphasized that such concept is undermined by the presence of “uncommon” hybrid fibres in control PL and, on a broader level, in normal adult skeletal muscle [28]. An alternative hypothesis is that polymorphic fibres contribute to a “fine-tuning” of muscle activity by ensuring efficient functioning at varying shortening speeds [4, 28, 48]. From this perspective, the 4-wk immobilized PL can be considered as a “specialized” muscle compared with control PL. Interestingly, it has been shown that the optimal length of fast motor units (MUs) in PL is closer to the optimal length of whole muscle compared to slow MUs [49]. In the present model of immobilization at the optimal length of skeletal muscle, the fast MUs, and thus fast-MHC fibres, are prone to work nearer their optimal length in comparison with slow MUs. Since excursion is a major determinant of muscle plasticity [24, 50], it can therefore be assumed that the reduction in length variations in immobilized PL induces a specialization of muscle fibres toward pure–fast- MHC types. As a result, the 4-wk immobilized PL is tailored to work optimally at a specific length.

### P38 and JNK Phosphorylations, but Not ERK1/2, Are Upregulated by Immobilization in Peroneus Longus

A large body of evidence indicates that the MAPK cascades play a significant role in the transduction of mechanical signals into genomic responses and the regulation of MHC gene switching [18, 51–54]. The effects of ERK1/2 phosphorylation on muscle contractile phenotype are conflicting. Even though Shi et al. [18] have shown that the inhibition of ERK1/2 pathway induces a slower phenotype, the large majority of experiments in myoblasts and cultured myotubes have demonstrated that ERK1/2 activation upregulates MHC I transcription [51, 52, 54]. The ERK1/2-mediated phosphorylation of p300 could explain the fast to slow-twitch fibre phenotype transition by enhancing NFATc1 transactivation [54]. Interestingly, hindlimb unloading is recognized to decrease both ERK1/2 phosphorylation and MHC I isoform expression in muscle fibres [35], whereas electrostimulation prevents such modifications [35, 40]. In contrast to other models of disuse, we did not observe a reduction in ERK1/2 phosphorylation in immobilized PL, maybe due to the preservation of neural inputs in this model [13]. Finally, all these results suggest that ERK1/2 does not play a role in the decrease of MHC I proportion during isolated muscle immobilization.

In agreement with hindlimb unloading and cast-immobilization experiments [35, 55–57], we observed an increase in p38 and JNK phosphorylation in PL after 6 and 15 days of immobilization. In our model of immobilization at neutral length, the proximal and distal tendons of PL are fixed at the same bone (i.e. fibula; see Fig 2 in [13]). Accordingly, the contraction of the immobilized PL develops in an isometric fashion. It has been previously shown that isometric contraction increases p38 MAPK phosphorylation in isolated skeletal muscle preparation [16]. This activation of p38 and JNK can influence MHC expression through various cell signaling pathways. The MAPK-activated protein kinases 2 and 3 (MK2/3) represent protein kinases downstream of the p38 MAPK. Using MK2/3 double-knockout mice, Scharf and colleagues have demonstrated that silencing of these kinases induced an increase in MHC I expression in Soleus muscle [53], suggesting that p38 acts on contractile phenotype by inhibiting MHCI transcription activity. Moreover, it has been demonstrated *in vitro* that p38 and JNK directly phosphorylate NFATc1 serine residues and inhibit the translocation of transcription factor to the nucleus [20, 21]. The myocyte enhancer factor-2 (MEF-2) family is also a key factor for controlling MHC isoform expression in myocytes [58]. Using pharmacological inhibition in transgenic mice, Meissner and colleagues have demonstrated that p38 MAPK upregulates MHCIId/x gene expression through the recruitment of a transcriptional coactivator, the CREB-binding protein (CBP), at a proximal MEF-2 site [54]. Altogether, these data strongly suggest that the increase in p38 and JNK phosphorylation observed during single muscle immobilization could induce a slow-to-fast MHC transition through the modulation of NFATc1 and MEF-2 transcription activity.

### Study Limitations

We acknowledge some limitations in this study. First, we compared the effects of muscle immobilization to the contralateral muscle, rather than using muscles from non-operated animals. To our knowledge, there has been so far little discussion about this key methodological issue, namely the use of the contralateral muscle as a pertinent control in animals submitted to unilateral immobilization. One can imagine that ipsilateral immobilization would induce a mechanical compensation in contralateral muscle leading to hypertrophy, or the opposite, a decrease in animal activity with subsequent alterations in skeletal muscle [59]. However, these concerns have not been substantiated by experimental data from studies using both control and contralateral muscles [9, 59, 60]. It should be emphasized that muscles from the non-operated limb are unaffected during unilateral immobilization, including when the immobilization procedure involves a whole joint, and thus several muscles [9, 59, 60]. Further, in preliminary studies, we have checked that there were no differences in muscle weight and index of MHC polymorphism between control PL from non-operated animals and contralateral non-immobilized PL.

The respective roles of p38 and JNK in the modulation of single-fibre myosin heavy chain polymorphism also need to be confirmed in further experiments. For this purpose, the use of pharmaceutical inhibitors intraperitoneally injected (e.g. anisomycin for p38, SP600125 for JNK) or genetically modified mice models (e.g. p38 or JNK mouse models) constitute promising approaches to explore the role of these MAPK during muscle immobilization.

### Conclusion

In summary, this study demonstrated that single muscle immobilization at neutral length induces a shift of MHC composition toward a faster contractile phenotype and decreases the polymorphic profile of single fibres. Finally, activation of p38 and JNK could be a potential mechanism involved in these contractile phenotype modifications during muscle immobilization. Altogether, these findings finally provide new insights to better understand whether and how contractile phenotype is modulated during bed rest, casting or other muscle-debilitating conditions.

## Supporting Information

S1 File Dataset. (XLS)

S1 Fig MAP kinase activation in PL from operated and contralateral hindlimbs. MAP kinases phosphorylation in sham, 6- and 15-days immobilized PL (n = 4 animals in each condition). AU, arbitrary unit. Data are expressed as mean ± SD. * MAPK activation significantly different from contralateral muscles (*P* < 0.05), # fold-change significantly different from sham-operated group (*P* < 0.05).
(TIF)

## Abbrevations

ERK: Extracellular signal-Regulated Kinases
JNK: *c*-Jun- NH2-terminal kinase
MAPK: Mitogen-Activated Protein Kinases
MHC: Myosin Heavy Chain
p38: protein kinase 38
PL: Peroneus Longus
SDS: PAGE Sodium Dodecyl Sulfate PolyAcrylamide Gel Electrophoresis
